# Performance of community health volunteers during the COVID-19 pandemic: assessing the enablers and challenges in Machakos County, Kenya

**DOI:** 10.1017/S1463423625100248

**Published:** 2025-07-30

**Authors:** Ann Wanyaga Mwaniki, John Muge Nyaboga, Ezekiel Onyonka Mecha, Boniface Oyugi

**Affiliations:** 1 County Government of Machakos, Department of Health Services, County Government of Machakos, Machakos Highway, P.O Box 2574-90100, Machakos, Kenya; 2 Department of Biochemistry, University of Nairobi, 30197-00100, Nairobi, Kenya; 3 M and E Advisory Group, P.O. Box 6523-00200, Western Heights, The Mint Nairobi, Kenya; 4 Centre for Health Services Studies (CHSS), University of Kent, George Allen Wing, Canterbury CT2 7NF, UK; 5 Department of Nursing Sciences, University of Nairobi, 30197-00100, Nairobi

**Keywords:** Challenges, community health volunteers, COVID-19 pandemic, enablers, Kenya

## Abstract

**Aim:**

This study explored the enablers and challenges influencing the performance of community health volunteers (CHVs) in Machakos County, Kenya, during the COVID-19 pandemic.

**Background:**

The COVID-19 pandemic disrupted healthcare systems globally, with particularly severe impacts in developing countries. Community health workers (CHWs) played a critical role in crisis communication, community engagement, case detection, referrals, and maintaining care continuity. However, limited evidence exists on the factors enabling and hindering their performance during the pandemic.

**Methods:**

This study employed a convergent mixed-methods design, integrating focus group discussions (FGDs), in-depth interviews (IDIs), and structured data extraction from the Kenya Health Information System (KHIS). Analysis of the data was guided by Agarwal et al.’s conceptual framework for measuring community health workforce performance with the quantitative data being analyzed using descriptive statistics, while qualitative data being analyzed through thematic analysis.

**Findings:**

CHVs effectively disseminated COVID-19 information, addressed vaccine hesitancy, and mobilized communities, supported by training, supervision, and community recognition. Their efforts led to significant improvements in healthcare services, including increased household visits, immunizations, and maternal health referrals. Despite their contributions, CHVs faced challenges such as delayed stipends, limited resources, and occasional community stigma, which hindered performance. Social support networks, community appreciation, and priority healthcare access emerged as key enablers, fostering resilience and motivation. Improved reporting mechanisms also highlighted CHVs’ expanded roles during the pandemic.

**Conclusion:**

This study underscores the critical role of CHVs in sustaining healthcare services during the COVID-19 pandemic, despite facing financial, logistical, and social barriers. Their resilience and adaptability led to significant improvements in key health services, supported by effective supervision and training. Strengthening systemic support, integrating CHVs into long-term strategies, and enhancing community recognition are essential to maximize their impact in future health challenges.

## Introduction

Kenya has made significant progress in institutionalizing community health services at both policy and practice levels, aligning with global shifts such as the 2012 United Nations declaration on universal health coverage (UHC) and the renewed emphasis on primary health care through the Alma Ata Declaration of 1978 and the Astana Declaration of 2018 (Hussein *et al.*, 2021). Since its adoption in 2006 under Kenya’s Second National Health Sector Strategic Plan (NHSSP II: 2005-2010), the Community Health Strategy (CHS) has evolved significantly (Kenyan Ministry of Health, 2021). Originally aimed at promoting proactive community and individual health to reduce disease burdens, the strategy has since become a cornerstone of Kenya’s health framework (Kenya Ministry of Health, 2020). CHS has been integrated into key policies, including the Kenya Vision 2030 and the Health Act of 2017, emphasizing its importance in achieving UHC (Kenyan Ministry of Health, 2021, Kenya Ministry of Health, 2020). Further advancements include the establishment of Community Health Units (CHUs) as the first level of healthcare and the development of comprehensive guidelines like the Kenya Community Health Policy (2020–2030) and the Kenya Community Health Strategy (2020–2025) (Kenya Ministry of Health, 2020, Kenyan Ministry of Health, 2021). These frameworks focus on strengthening governance, workforce capacity, financing, and integration with primary healthcare to address both persistent and emerging health challenges.

Community health workers (CHWs) are vital to health systems serving as key links between communities and formal healthcare and recognized as essential for delivering effective and equitable health services (Salve *et al.*, 2023, Zulu and Perry, 2021, Ogutu *et al.*, 2023). Known by various names depending on context–such as community distributors, health promoters, village health workers, barefoot doctors, lay health workers, traditional birth attendants, and lay counsellors–CHWs in Kenya are commonly referred to as community health volunteers (CHVs) (Hussein *et al.*, 2021, Ogutu *et al.*, 2023). However, on September 25, 2023, the Kenyan government launched the Community Health Promoters (CHPs) programme, shifting focus from curative to community and primary healthcare, providing CHPs with tools, digital resources, and remuneration to advance UHC. While the name CHVs was updated to CHPs, this paper uses the term CHVs to align with the study’s timeline and context of the data collection time.

Evidence from low and middle income countries (LMICs) highlights the critical role of CHVs in enhancing access to primary healthcare services, including maternal and neonatal care, mental health support such as counseling for anxiety and depression, and preventive education on hygiene, sanitation, exclusive breastfeeding, and family planning (Medhanyie *et al.*, 2012, Kimani-Murage *et al.*, 2016, Baker-Henningham *et al.*, 2005, Ali *et al.*, 2003, Sheth and Obrah, 2004, Nankunda *et al.*, 2006, Haider *et al.*, 2000, Scott *et al.*, 2015). CHVs address community health needs, particularly among vulnerable groups such as women, children, the elderly, and the disabled (Olaniran *et al.*, 2017). They also capture epidemiological data, route consultations, guide patients on long-term medications, and support immunization and vector-control initiatives (Owais *et al.*, 2011). During health emergencies like the Ebola outbreak in West and Central Africa and the COVID-19 pandemic, CHVs were pivotal in crisis communication, community engagement, early case detection, contact tracing, and facilitating referrals for testing and continued care (Hartzler *et al.*, 2018, Africa Centres for Disease Control and Prevention, 2021, Miller *et al.*, 2018). These contributions make CHVs essential to improving health outcomes in resource-constrained settings.

**Figure 1.**
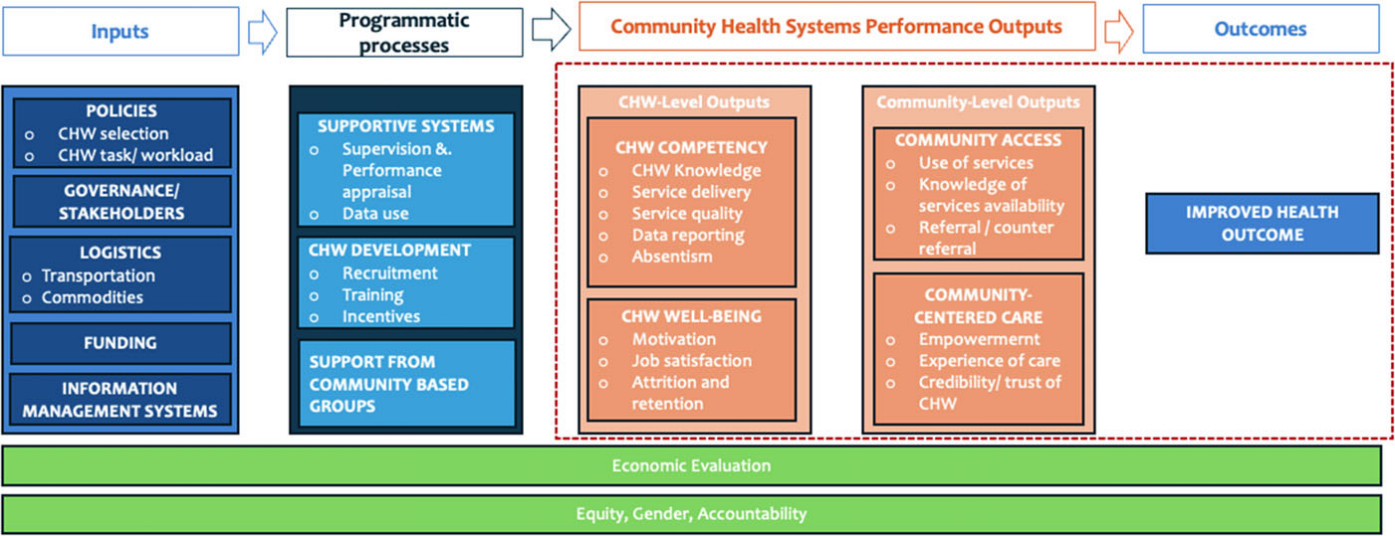
Community health worker performance measurement framework.

Agarwal *et al.*’s (2019) conceptual framework for measuring community health workforce performance within primary healthcare systems offers a comprehensive structure for evaluating the contributions of CHVs, encompassing four interconnected domains: inputs, programmatic processes, outputs, and outcomes. This study was informed by the framework proposed by Agarwal *et al.*’s (2019) (Figure 1). According to Agarwal and colleagues, inputs include the enabling environment necessary for CHV programmes, such as policies, governance, logistics, financing mechanisms, and information management systems. Programmatic processes focus on operational components, including support systems (e.g., supervision, performance appraisal, and data use), CHV development (e.g., recruitment, training, and incentives), and community engagement to ensure CHVs are well-prepared and motivated. The framework defines outputs at both the CHV and community levels. At the CHV level, it evaluates competence (knowledge, service delivery, quality of care, data reporting, and absenteeism) and well-being (motivation, job satisfaction, attrition, and retention). At the community level, it assesses access (service use, knowledge of service availability, and referral processes) and community-centred care (empowerment, care experience, and trust in CHVs). Finally, outcomes capture broader impacts, including improved health service utilization, equity, and overall health outcomes, emphasizing the importance of CHVs in strengthening primary healthcare systems. This study focused on outputs at both the CHV and community levels and outcomes, as emphasized in Figure 1.

Literature highlights that the performance of CHVs is highly influenced by the context in which they work, including factors related to their specific settings, the broader environment and the time period of work (Kok *et al.*, 2015). Understanding the timing and contextual factors affecting CHV performance is essential for optimizing their effectiveness. In supporting Agarwal *et al.*’s (2019) framework, studies across various contexts reveal numerous challenges in the delivery of primary health services by CHVs, including logistical issues like inconsistent reporting and lack of real-time data, resource limitations such as inadequate training and remuneration, high workloads, insufficient supervision, and cultural and financial barriers (DeRenzi *et al.*, 2017, Jerome and Ivers, 2010, Brunie *et al.*, 2014, Aseyo *et al.*, 2018, Kuule *et al.*, 2017). However, these reviews were conducted in the context prior to the COVID-19 pandemic.

The COVID-19 pandemic placed unprecedented strain on global healthcare systems, significantly disrupting routine and essential services delivered by CHWs (Bezbaruah *et al.*, 2021). The COVID-19 pandemic exposed systemic vulnerabilities in community healthcare delivery, significantly affecting CHVs’ ability to implement health initiatives. It underscored the need to examine the enablers and challenges influencing CHVs’ performance during the crisis, despite their critical role in delivering essential healthcare services.

Consequently, this study sought to explore the enablers and challenges influencing the performance of CHVs during the COVID-19 pandemic in Machakos County, Kenya. CHVs are essential in pandemic responses, requiring clear roles, adequate training, supportive supervision, and overall well-being to ensure effectiveness. Their integration into health emergencies is vital to strengthen system resilience and disaster preparedness (Wiig and O’Hara, 2021, World Health Organization, 2018). Therefore, there is a necessity for more investigation of CHWs in pandemics (Bhaumik *et al.*, 2020). The COVID-19 unclear trend line resulted in substantial challenges for healthcare providers (Lusambili *et al.*, 2021, Ness *et al.*, 2021, Razu *et al.*, 2021). Due to the vital part of these healthcare providers in the pandemic reaction, this study aimed to achieve an enhanced grasp of the challenges of CHVs during the COVID-19 period and the enablers and their performance during the pandemic.

## Methods

### Study design

**Figure 2.**
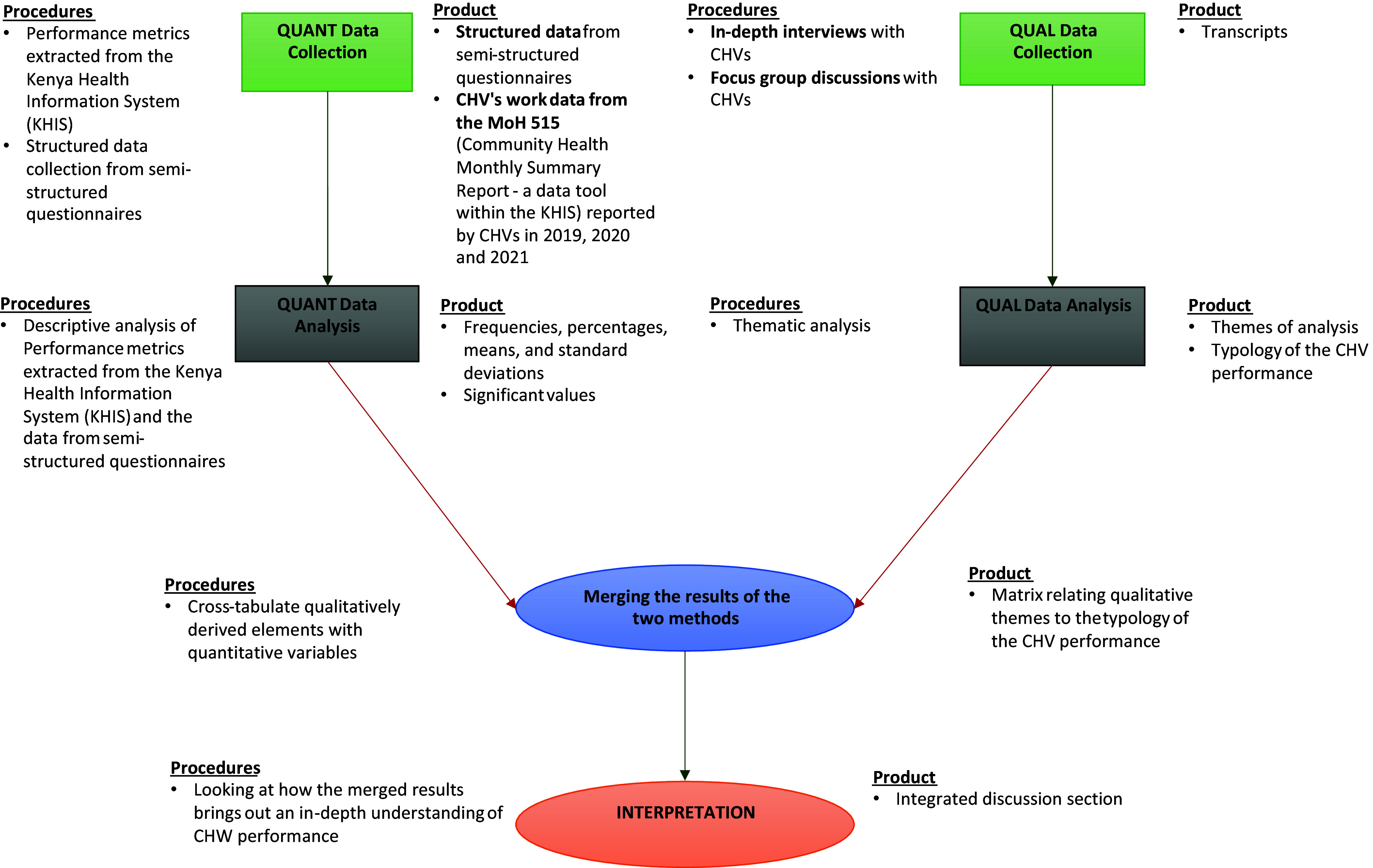
Study design and processes.

This study employed a convergent mixed-methods research design, utilizing a fully integrated approach to comprehensively explore CHVs’ work output and experiences (Creswell and Clark, 2017). Both qualitative and quantitative data were collected concurrently at multiple points to capture the complexity of CHVs’ performance and provide a holistic understanding of their roles. Quantitative data included performance metrics extracted from the Kenya Health Information System (KHIS) and structured data from semi-structured questionnaires administered during in-depth interviews (IDIs) with CHVs. Simultaneously, qualitative data were obtained through open-ended questions in the semi-structured questionnaires during IDIs and through focus group discussions (FGDs). The integrated analysis allowed for the synthesis of quantitative findings with qualitative insights, offering a nuanced understanding of the roles, challenges, and enablers affecting CHVs in the study setting. Design summary is in Figure 2.

### Study site

This research was conducted in Machakos County, located in Eastern Kenya. The county is bordered by Nairobi and Kiambu Counties to the west, Embu County to the north, Kitui County to the east, Makueni County to the south, and Kajiado, Murang’a, and Kirinyaga Counties to the southwest and northwest. Machakos consists of nine sub-counties: Machakos Township, Mavoko, Kathiani, Masinga, Yatta, Matungulu, Mwala, Kangundo, and Kalama. According to the 2019 census, the county has a population of approximately 1,421,932, with over two-thirds (70.9%, or 1,007,854 people) residing in rural areas (Kenya National Bureau of Statistics, 2019). For this study, four sub-counties were purposively selected based on population characteristics: Machakos Township (170,606) and Athi River (322,499) representing urban populations, and Masinga (148,522) and Yatta (172,583) representing rural populations (Kenya National Bureau of Statistics, 2019, Kenya Commission on Revenue Allocation, 2022). Machakos Township was chosen as the county headquarters, where all COVID-19 resources were centrally managed, while Athi River, a densely populated sub-county bordering Nairobi, was included due to its heightened disease surveillance activities linked to its proximity to the capital (Gitau *et al.*, 2021, Kenya Human Rights Commission, 2020). Further, Masinga and Yatta, representing rural areas, were selected due to their higher poverty levels of 26.1% and 27.2%, respectively, in contrast to the more urban Machakos Township (21.6%) and Athi River (18.3%) (Kenya Commission on Revenue Allocation, 2022). This selection enabled the understanding of the CHVs capacity to perform their roles effectively in urban and rural areas. Mwala, despite having the highest poverty level (33.6%) and the second-largest population (181,896), was excluded due to accessibility challenges, limited timelines, and budget constraints.

### Study population, selection procedure and sampling

The study focused on CHVs actively working during the COVID-19 outbreak who consented to participate. At the time of the study, there were 1,960 CHVs in Machakos County (Kenya Health Information System, 2021). CHVs who were on leave, unwell, had less than one year of service without prior CHV training, or were away for training or illness were excluded from the study. The participants were engaged through IDIs and FGDs. The rationale for employing both IDIs and FGDs was to gain a comprehensive understanding of CHVs’ experiences and perspectives by collecting both individual and group insights. This approach was particularly relevant given that CHVs frequently collaborate in groups during community action days and health-related activities. The IDIs provided an opportunity for in-depth exploration of personal experiences, while the FGDs captured collective dynamics, shared viewpoints, and interactions within group settings. This dual methodology ensured a nuanced and holistic understanding of the CHVs’ roles, challenges, and contributions during the pandemic.

Sampling for the study was conducted in two distinct forms: one for IDIs and the other for FGDs. For the IDIs, the sampling was calculated based on the 1,960 CHVs active in Machakos County at the time of the study. Among these, the four sub-counties selected for the study had a total of 960 CHVs, distributed across 96 CHUs with 10 CHVs in each CHU (Table 1). A sample size of 282 CHVs from the 960 was estimated using Yamane’s (1967) formula for determining sample size, as follows (Tepping, 1968):

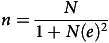




**Table 1.** Proportionate sampling

Sub-county	CHUs	CHVs	Sample Proportion
Athi River	10	100	100/960*282=29
Machakos	23	230	230/960*282=68
Masinga	35	350	350/960*282=103
Yatta	28	280	280/960*282=82
**Total**	**96**	**960**	**282**

Where n = sample size; N = the population size (960); e = the level of precision at a 95% confidence level, and p = 0.5. Proportionate sampling was then applied to allocate the sample size across the sub-counties. The number of respondents from each sub-county was determined using the number of CHUs and CHVs within each sub-county as the sampling frame Table 1. None of the participants withdrew or dropped out. For the FGDs, the CHVs who had not participated in the IDIs were purposively selected. The selection of participants for the FGDs was based on their shared experience in providing COVID-19-related support and their availability to participate in the scheduled discussions. To ensure homogeneity, factors such as gender, age, experience, and education levels were carefully considered. Ninety per cent of themes can typically be identified within 3 to 6 FGDs, with three FGDs often being sufficient to capture the most prevalent themes (Guest *et al.*, 2017). However, in this study, three FGDs were conducted as new and relevant information continued to emerge, particularly on topics such as CHVs’ access to organizational resources, the challenges they faced, and their pressing difficulties. Each FGD consisted of 5 to 12 participants, ensuring diverse viewpoints while maintaining manageable group sizes to facilitate in-depth discussions.

Sampling was not required for the extraction of quantitative performance metrics, as the data was directly retrieved from the KHIS.

### Data collection tools and procedure

As mentioned earlier, the study employed IDIs, FGDs, and data extraction from the KHIS. All the data were collected between September and December 2017. For the IDIs, a semi-structured questionnaire was designed, drawing from previous studies (Kok *et al.*, 2015, Kok *et al.*, 2017, Kuule *et al.*, 2017, Vareilles *et al.*, 2015) and tailored to address the research questions. The questionnaire captured respondents’ socio-demographic characteristics, providing insights into which groups were more actively involved in community health, such as gender (male or female) and age brackets (older or younger individuals). Additionally, it explored participants’ work experience, output, challenges faced during the COVID-19 pandemic, and the enablers that facilitated their work during this period.

The second tool, an FGD guide, was developed to address gaps identified from the IDIs as guided by other researchers (Guest *et al.*, 2017, Bakibinga *et al.*, 2020, Yansaneh *et al.*, 2016), and captured gaps in CHVs’ access to organizational resources, recognition of their efforts, and the value placed on their input during the pandemic. It also delved into the challenges CHVs faced and their most pressing difficulties. This tool complemented the data gathered through IDIs, allowing the researcher to probe more deeply and obtain richer, more nuanced insights.

The third section focused on CHVs’ work output, utilizing data from the MoH 515 (Community Health Monthly Summary Report) (Kenyan Ministry of Health, 2022), a tool within the KHIS, for the years 2019, 2020, and 2021. This report documented CHV activities such as household visits, health insurance coverage, and access to safe water and sanitation. It also tracked reproductive health indicators, including family planning counselling, antenatal care (ANC) referrals, and postnatal care, as well as nutrition metrics like malnutrition screening and immunization for children under five. HIV and TB services were monitored through data on testing, treatment referrals, and defaulter tracing. Additionally, the tool captured chronic illness referrals (e.g., diabetes and hypertension), community activities, and reported deaths.

The study tools, including the semi-structured interview guide and the FGD guide, were pre-tested (by the first author) among CHVs in Mwala sub-County, a non-participating sub-county, to ensure their validity and reliability. Validity was assessed with input from the supervising lecturers (LWKB and ACK), experts in the field, who evaluated the tools’ content to confirm its relevance and representation of the study subject. Reliability was established through a test-retest procedure, conducted two days apart, to ensure consistency and stability in the tools’ performance.

The qualitative interview guides were developed in English, translated into Swahili, and back-translated to ensure accuracy and quality when compared to the original English version. These tools, including the semi-structured interview guide and the FGD guide, were pre-tested among CHVs in the Mwala Sub-county of Machakos County, a non-participating sub-county. This pilot testing ensured the validity and reliability of the tools. Validity was established through evaluation by supervising lecturers, who assessed the content to confirm it was representative of the study objectives. Reliability was evaluated using a test-retest procedure conducted two days apart, and the guides were revised based on the outcomes of the pilot study.

Before the commencement of the study, data collectors, who were unfamiliar to the respondents, briefed participants about the study objectives. Participants were provided with detailed information about the organization conducting the study, the purpose, and the researchers, after which they gave written informed consent. The IDIs were conducted by the first author (AWM), with the support of two trained research assistants who had experience in qualitative research, community work, and fluency in English and Swahili. The research assistants were trained on the project objectives, study guides, informed consent procedures, and interviewing techniques. Each IDI took approximately 45 to 60 minutes and was conducted in private settings to ensure confidentiality. Similarly, the FGDs were facilitated by AWM in Swahili, assisted by an observer (a public health officer (PHO)) and a note-taker (a research assistant). Each FGD lasted between 30 and 45 minutes and were held in private spaces to encourage participants to express themselves freely. Observational data, including non-verbal cues were captured during both the FGDs and IDIs.

As the data collection was conducted during the height of the COVID-19 pandemic, strict adherence to Kenyan MoH guidelines was observed. Measures included maintaining social distancing of 1.5 metres, wearing face masks properly, and practising hand hygiene throughout the data collection process. All interviews and discussions were audio recorded and conducted in spaces free from distractions, eavesdropping, and external pressures.

Quantitative data on CHVs’ work output was abstracted by AWM from the KHIS for the period of January 2019 to December 2021. This process involved reviewing the County’s MoH 515 monthly community health summary reports, which documented various performance metrics reported by CHVs. This comprehensive approach ensured that both qualitative and quantitative data were collected with rigour and reliability, providing valuable insights to meet the study’s objectives.

### Data analysis

Quantitative data, derived from the structured questions and the MoH 515 form, was entered and cleaned in Excel before being analyzed using the Statistical Package for Social Sciences (SPSS version 25.0). The analysis employed descriptive statistics, including frequencies, percentages, means, and standard deviations, to summarize the findings.

Qualitative data, derived from the open-ended questions in the questionnaires and FGDs, was transcribed verbatim by AWM, with transcription accuracy verified by reviewing the transcripts against the audio files and cleaning them where necessary. All transcripts, field notes, and electronic documents were uploaded into NVivo 12 Pro software (QSR International) for effective organization and management during data analysis. Thematic analysis followed the six-step approach by Clarke and Braun (2017). In step one, the interviews were transcribed verbatim and repeatedly reviewed by AWM to ensure familiarity with the data. In step two, initial codes were generated by AWM and the supervising lecturers, guided by the elements of the study’s conceptual framework. This collaborative approach aimed to enhance the consistency and credibility of the coding process. Any discrepancies in coding were discussed and reconciled before finalizing the codes. In step three, the finalized codes were collated into potential themes, and a theme map was created to ensure that the identified themes aligned with the coded extracts and the overall dataset. Step four involved reviewing and refining the themes, ensuring they accurately reflected the data. In step five, definitions and names for the themes were established through further discussion. Finally, in step six, the qualitative findings related to CHAs were synthesized and integrated with the quantitative results, providing a holistic understanding of the study’s outcomes.

### Trustworthiness and reflexivity

To ensure the trustworthiness of the study findings, we employed multiple data collection approaches (method triangulation), engaged diverse CHV participants with different characteristics to capture various perspectives (data source triangulation), utilized iterative questioning by rephrasing questions and using probes, and conducted peer debriefing sessions with the study team.

Three of the four authors are registered nurses with training and experience in community health work, including roles similar to those of CHWs and CHVs across various settings in Kenya. Notably, one author has worked directly with communities in the study county, which shaped their interest in the research topic and informed key methodological decisions, such as participant sampling, data collection approaches, and analysis strategies.

## Results

A response rate of 100% was attained from all the 282 CHVs in the IDIs. Two FGDs were conducted among select CHVs. Most of the study participants were female (78%, n = 220); aged 30 years and above (83%, n = 234); were married (84.8%, n = 239); had basic education background (Primary Completed - 21.3%, n = 60; Secondary Incomplete - 26.2%, n = 74; Secondary Completed - 40.1%, n = 113) and had worked as CHVs for over 5 years (78%, n = 220) (Table 2). Similarly, majority of the FGDs participants were female CHVs, aged between 33 and 58 years, most of whom were also married and had basic education background.

**Table 2.** Summary of socio-demographic characteristics of the respondents, and enablers and challenges of CHVs work

Socio-demographic characteristics of the respondents		Frequency (n = 282)	Percent
Gender	Male	62	22.0
	Female	220	78.0
Age	20 – 29 years	5	1.8
	30 – 39 years	43	15.2
	40 – 49 years	112	39.7
	50 years and above	122	43.3
Marital status	Single	17	6.0
	Married	239	84.8
	Widowed/Separated/Divorced	26	9.2
Education level	Primary Incomplete	19	6.7
	Primary Completed	60	21.3
	Secondary Incomplete	74	26.2
	Secondary Completed	113	40.1
	Tertiary	16	5.7
Duration worked as a CHV	6 months or less	1	0.4
	1 – 5 years	61	21.6
	Over 5 years	220	78.0
**Enablers**			
Having priority access to health services	Yes	13	4.6
	No	269	95.4
Belonging to a Social Support Group	Yes	240	85.1
	No	42	14.9
Efforts Appreciation by the Community	Yes	280	99.3
	No	2	0.7
Social support from the community	Yes	32	11.3
	No	250	88.7
Monetary compensation for the CHV role	Yes	274	97.2
	No	8	2.8
**Challenges during the COVID-19 pandemic**			
Experienced inadequate or delayed stipend	Yes	282	100
	No	0	0
Experienced social stigma in their work	Yes	137	48.6
	No	145	51.4
Had concerns about contracting COVID-19	Yes	282	82.3
	No	50	17.7
Faced community opposition in their work	Yes	84	29.8
	No	198	70.2
experienced inadequate or unsupportive supervision	Yes	11	3.9
	No	271	96.1
experienced inadequate on-job training	Yes	244	86.5
	No	38	13.5
Suffered excessive workload	Yes	255	90.4
	No	27	9.6
Experienced difficulty in transport	Yes	267	94.7
	No	15	5.3
Experience lack of tools and materials	Yes	260	92.2
	No	22	7.8

### Community health systems performance outputs and outcomes

This section underscores the outputs and outcomes derived from the CHWs Performance Measurement Framework at both the CHV and community levels. CHVs and their communities were pivotal during COVID-19, effectively raising awareness, combating vaccine hesitancy, and enhancing health outcomes despite facing significant challenges such as excessive workloads, delayed compensation, and limited resources. Their dedicated efforts contributed to improvements in immunization coverage and antenatal care. However, issues like social stigma and a lack of recognition from peers and health systems reveal the pressing need for enhanced systemic support.

#### CHW competency

*The CHVs demonstrated competence in raising awareness about COVID-19 protocols within their communities.* They successfully taught and demonstrated key public health and social measures, such as proper mask-wearing, handwashing with soap and water, and maintaining social distance. These efforts were carried out through various avenues, including home-to-home visits, public gatherings like chiefs’ barazas, and local churches. Additionally, CHVs actively monitored community adherence to these protocols, ensuring their effective implementation.

*“We were able to teach community members about COVID 19 prevention and particularly on how to wear masks and how to do proper hand washing. We also moved round homes to observe community members’ application of the guidelines.” **– Participant 004, FGD 01***

*“We mobilised community members into Barazas and educated them about COVID-19 and measures to protect oneself from the infection.” **– Participant 003, FGD 02***

*CHVs showcased their competence in addressing myths and misconceptions surrounding the COVID-19 vaccine and effectively mobilizing community members to receive vaccinations.* They educated the public on how the vaccine works, its safety, and the importance of vaccination. By addressing concerns and answering questions, CHVs helped dispel fears, foster vaccine acceptance, and contributed to increasing the number of people vaccinated against COVID-19.

*“We urged our fellow community members not to fear the COVID-19 vaccines and encouraged them to get vaccinated against the COVID-19 infection.” **– Participant 008, FGD 01***

*Supportive supervision enhanced CHV competencies.* Supportive supervision played a key role in enhancing the competencies of CHVs during the COVID-19 pandemic. A majority of respondents (96.1%, n = 271) reported receiving adequate supervision, which provided essential guidance and assistance in their fieldwork, enabling them to perform their roles effectively. However, a small minority highlighted challenges with inadequate or unsupportive supervision, citing instances of being left to work alone for extended periods. This lack of support negatively impacted their effectiveness and morale, underscoring the importance of consistent and hands-on supervision.

*Adequate on-the-job training and collaboration with various stakeholders during the COVID-19 pandemic significantly enhanced CHV performance.* Adequate on-the-job training during the COVID-19 pandemic played a pivotal role in enhancing CHV performance. A majority of respondents (86.5%, n = 244) reported receiving sufficient training, which prepared them to effectively address the challenges of the pandemic. However, a minority highlighted inadequacies in the training, citing limited sessions, short durations, and insufficient content coverage, emphasizing the need for more robust capacity-building initiatives.

CHVs attributed their success during the pandemic to three key factors: unwavering dedication to their work despite numerous challenges, effective teamwork and collaboration with community stakeholders, and training on COVID-19 prevention protocols. One CHV noted, *“I remained dedicated to my work throughout the COVID-19 pandemic. I am also grateful that we had undergone training on COVID-19 prevention protocols” **(Participant 007, FGD 01).*** Another emphasized the role of collective efforts, stating, *“Our success was rooted in teamwork and close collaboration with various key players in our communities including government and religious leaders” **(Participant 006, FGD 02).*** These factors underscore the resilience and commitment of CHVs in navigating the pandemic’s demands

*Excessive workload impeded service delivery.* The majority of respondents (90.4%, n = 255) reported experiencing excessive workloads during the COVID-19 pandemic, which significantly hampered their ability to deliver services effectively. This workload stemmed from multiple responsibilities, including raising awareness and educating communities about COVID-19 prevention protocols, regularly tracking and following up with COVID-19 victims, conducting extensive household visits to promote adherence to prevention measures and vaccine uptake, and compiling numerous reports related to COVID-19 activities. These cumulative demands placed a considerable strain on CHVs, challenging their capacity to maintain efficient service delivery.

#### CHW well-being

*Challenges in monetary compensation and support for CHVs.* Nearly all respondents (97.2%, n = 274) confirmed receiving monetary compensation in the form of a stipend provided by the County Government for their roles as CHVs. However, all respondents (100%, n = 282) reported significant issues related to these stipends, citing inadequacy, irregular payments, and prolonged delays. The insufficient amounts and extended waiting periods, sometimes stretching to months or even years, severely impacted their financial stability and morale. One CHV expressed their frustration:

*“The pay we receive is very little and is often delayed. It could be months and, at times, years before the stipend arrives. Yet you’ve been serving faithfully all through. That’s our predicament” **(Participant 007, FGD 01).***

In addition to financial challenges, CHVs perceived a lack of adequate support and recognition from the government during the COVID-19 pandemic. This sense of neglect was compounded by unrealistic expectations from the impoverished communities they served, where CHVs were often expected to provide food and medication, needs far beyond their mandate. The widespread poverty in these communities further exacerbated the situation, leaving many CHVs unable to afford basic necessities like food and water while on duty, leading to hunger and thirst during their workdays. These compounded challenges highlighted the pressing need for better financial support, government recognition, and realistic expectations to sustain CHVs in their critical roles.

*Many CHVs voiced frustration over insufficient government support, highlighting not only delays and inadequacy in compensation but also the lack of essential tools and logistical resources needed to perform their duties effectively.* These challenges were exacerbated during the COVID-19 pandemic, which increased living costs, transport expenses, and operational demands, such as the need to procure personal protective equipment (PPE) like masks and sanitizers. The majority (92.2%, n = 260) of respondents reported facing a significant shortage of tools and materials during the pandemic. CHVs cited a lack of adequate face masks, uniforms, gloves, and sanitizers. In some cases, they were provided with first aid kits and CHV bags that were either empty or poorly equipped. Insufficient reporting tools also posed a challenge, as CHVs often had to spend their own money on photocopying necessary forms—a burden they deemed unfair. These resource gaps not only hindered their work but also contributed to their sense of being undervalued. Some respondents remarked:

*“Sometimes we are given empty CHV bags without necessary tools and materials needed for our work, and no explanations are offered.” **– Participant 007, FGD 01***

*“Even during the COVID-19 pandemic, we were not provided with protective materials. We lacked masks, gloves and even the first aid kits given were empty. You are even forced to use your own airtime to do client’s follow-ups with false promises that this would be compensated, but it’s never reimbursed.” **– Participant 004, FGD 02***

*“The government does not provide us with the necessary tools and materials required for our work. They also do not facilitate our movement and communication with the communities we serve” **(Participant 003, FGD 01).***

These challenges highlight the significant financial and logistical burdens CHVs face, reinforcing the urgent need for systemic reforms to guarantee timely payments, sufficient stipends, and comprehensive operational support. Addressing these issues is critical to sustaining CHVs’ vital contributions to community health systems and enhancing their capacity to serve their communities effectively.

*CHVs experienced some social stigma and concerns about contracting COVID-19.* The findings revealed that slightly over half (51.4%, n = 145) of CHVs reported not experiencing social stigma in their work, while the remaining respondents acknowledged facing stigma. For those who experienced stigma, the issues stemmed from community members’ fears and misconceptions, such as the perception that CHVs might infect them with COVID-19. Additional factors included community members looking down on CHVs, ignoring them, displaying hostility, or avoiding interactions altogether.

Furthermore, a significant majority (82.3%, n = 232) of respondents expressed concerns about contracting COVID-19 during their work. These concerns were primarily attributed to a lack of adequate PPE, including insufficient masks and sanitizers. The risk was heightened by their frequent interactions with unmasked individuals during community outreach activities. These challenges underscore the dual burden CHVs faced, balancing their critical roles in community health with personal health risks and social stigma.

### Community access

*Success in linking and referring patients to health facilities.* CHVs demonstrated success in linking and referring patients requiring advanced care to local healthcare facilities. They ensured that cases beyond their capabilities were promptly referred for further management by specialists, minimizing missed opportunities for testing and specialized care. This proactive approach ensured patients received the necessary treatment, even when CHVs had to use their personal finances to facilitate referrals, showcasing their dedication to community health.

*“I remember a referral we had to do and had to call my fellow CHV because the patient was very sick and we managed to take the patient to the hospital. We had to use our money to get transport.” **– Participant 003, FGD 02***

*Transport challenges faced by CHVs during the COVID-19 pandemic hampered access to the services.* The majority of CHVs (94.7%, n = 267) reported significant transport difficulties during the COVID-19 pandemic. These challenges were largely attributed to the lack of transport options, forcing them to walk long distances to reach households within their assigned areas. The absence of adequate support exacerbated their hardships, as they often worked under harsh weather conditions and without basic sustenance. As one respondent explained, *“Transport is a big problem. We are forced to walk long distances as the households are sometimes quite far apart. Many times on an empty stomach and without even water to drink, yet the weather here is harsh” **(Participant 003, FGD 02).*** Another participant noted the high cost of alternative transport options, saying, *“We walk for long distances even in harsh weather conditions, like now it is very sunny and you have to walk from house to house and the houses are not close to each other. When you have money you use a motorbike, but it’s expensive” **(Participant 002, FGD 01).*** These transport difficulties placed an additional burden on CHVs, affecting their ability to effectively perform their roles.

*Community challenges in accepting the COVID-19 vaccine.* While the majority of respondents (70.2%, n = 198) reported not facing opposition from the community during the COVID-19 pandemic, some CHVs encountered resistance to their work. This opposition was primarily linked to community members’ reluctance to accept the COVID-19 vaccine, denial of the virus’s existence, and misconceptions about the vaccine, such as beliefs that it acted as a contraceptive or could cause death. Additionally, some community members feared that CHVs might infect them with COVID-19, further complicating their efforts to promote vaccine uptake and health education. Some CHVs noted:

*“Sometimes we get opposition from the community especially because of hunger in the community and the ignorant community members.” – **Participant 007, FGD 01***

*“Some community members despise our work, to the point of looking down on us. Some people even said we were COVID-19 carriers.” **– Participant 002, FGD 02***

#### Community centred care

*CHV efforts appreciated by the community members but not peers or other cadres of HCWs:* Nearly all respondents (99.3%, n = 280) reported that their efforts were highly valued by their communities. Community members consistently demonstrated their appreciation for CHVs’ efforts by consulting them on health matters, welcoming them into their homes, expressing gratitude for their services, and notifying them of cases that required attention. Additionally, they actively cooperated with CHVs’ work, provided moral encouragement, and offered assistance during personal or family-related challenges, reflecting a strong sense of community support and engagement. For instance, one respondent shared, *“The community appreciates us and tells us we are doing very good work for them” **(Participant 006, FGD 01)***, while another noted, *“People in the community tell us that we are helping them. They are happy when we take Vitamin A, dewormer, and other health services to them” **(Participant 008, FGD 02).***

However, when it came to support from peers and healthcare workers, the majority of respondents (88.7%, n = 250) reported receiving little to no formal social support. Some CHVs expressed frustration that peers and healthcare workers did not always acknowledge their contributions or act on the information they provided. One respondent remarked,

*“Though healthcare workers listen to us, they don’t act on information and suggestions we give” **(Participant 002, FGD 01).***

A clear example of the lack of support from peers and healthcare workers was the limited priority access to health services for CHVs. The majority of CHVs reported not receiving such privileges, with only a small fraction (4.6%, n = 13) indicating that they were prioritized when seeking care, such as being exempt from queuing at healthcare facilities.

*A majority of the CHVs participated in the social support groups which created a source of assistance and collaboration:* A significant majority of CHVs (85.1%, n = 240) reported belonging to social support groups within their communities. These groups included self-help groups and merry-go-rounds (22.3%, n = 63), welfare groups (16%, n = 45), table banking initiatives (43.3%, n = 122), and community-based organizations (3.5%, n = 10). Notably, all CHVs (100%) who were part of these social support groups acknowledged that membership was beneficial, providing valuable assistance, collaboration opportunities, and a sense of shared purpose in addressing community challenges.

### Increased health outcomes

The findings indicated increased output indicators among CHVs. Structured data from the IDIs revealed that in the past month, a significant proportion of CHVs (62.4%, n = 176) reported visiting more than 20 households, while a majority (77.0%, n = 217) conducted two or more health education sessions. Additionally, 42.2% (n = 119) addressed more than one public gathering (Barazas) to provide education, 76.3% (n = 215) attended two or more CHV meetings, and 43.3% (n = 122) referred two or more clients for further care, as summarized in Table 3.

**Table 3.** Community health volunteer (CHV) work output indicators as reported by the respondents

Work output indicators		Frequency (n = 282)	Per cent (%)
Number of households visited last month	1 – 10	28	9.9
	11 – 20	78	27.7
	Above 20	176	62.4
Number of health education forums conducted last month	None ever	6	2.1
	Only one	59	20.9
	2 – 4	122	43.3
	5 and above	95	33.7
Number of *Barazas* addressed last month	None	49	17.4
	Only one	114	40.4
	2 – 4	103	36.5
	5 and above	16	5.7
CHVs meetings attended in a month	None ever	2	0.7
	Only once	65	23.0
	2 – 4	208	73.8
	5 and above	7	2.5
Number of clients you referred last month	None	60	21.3
	Only one	100	35.5
	2 – 5	91	32.3
	6 and above	31	11.0

Similarly, as retrieved from the County’s MOH CHVs Data Collection Sheet (MoH 515), records for 2019 to 2021 indicate that there were some improvements in the work output of CHVs working in the county over the period, though most of the entries for 2019 for the various indicators of the CHVs work output were missing or undocumented possibly due to poor reporting due to low emphasis on the work of the CHVs in the pre-COVID-19 period. However, there was improvement in work output reported in subsequent years as COVID-19 enhanced the role of the CHVs. For instance, the number of children aged 0-11 months referred for immunization rose from 289 in 2019 to 1,814 in 2021. The number of immunization defaulters referred rose from 15 in 2019 to 121 in 2021. The number of women aged 15 – 49 years provided with FP commodities rose from 3,691 in 2020 to 4,212 in 2021. The number of pregnant women counselled on ANC services from 406 in 2020 to 1,742 in 2021. An additional summary of all the outputs is shown in Table 4.

**Table 4.** Community health volunteers (CHVs) work output indicators as documented in the county’s MOH CHVs data collection sheet

	2019	2020	2021
**Work output indicators**	**Cases (n)**	**Cases (n)**	**Cases (n)**
Child 6–59 months referred for Vitamin A supplementation	–	1,941	10,428
Children of 0–11 months referred for immunization	289	735	1,814
Immunization defaulters referred	15	192	121
Number of children 12–59 months dewormed	887	11,000	3,396
Number of women 15–49 yrs counselled on FP methods	–	4325	9289
Number of women 15–49 yrs Referred for FP Services	–	765	2150
Number of women 15-–49 yrs provided with FP commodities	–	3691	4212
Number of pregnant women counselled on ANC services	–	406	1,742
Number of pregnant women Referred to health facility	250	518	1,064
Number of pregnant women counselled on ANC services	–	406	1,742
Number of New deliveries that took place in Health facility	245	303	505
Number of new mothers visited within 48 hrs of delivery	–	37	166
Number of newborns visited at home within 48 hours of delivery	1,246	3,087	159
Home delivery referred for Post Natal Care services	–	21	64
Total number of community Action Days held	–	43	366
Total number of community dialogue days held	–	19	111
Total number of community units monthly meetings held	–	72	228
Total number of households visited by CHVs in the month (New Visit)	–	23,949	35,825
Total number of households visited in the month by CHVs (Revisit)	–	12,146	23,497

Source: Machakos County MOH 515 Revised CHVs Records, 2019 – 2021.

## Discussion

This study set out to investigate the enablers and challenges affecting the performance of CHVs in Machakos County, Kenya, during the COVID-19 pandemic. Recognizing the critical role of CHVs in pandemic responses, the research focused on understanding the factors that enhanced or hindered their effectiveness in delivering community-based healthcare services. By identifying these key influences, the study sought to provide insights for strengthening CHV systems in future health emergencies.

The results highlighted the critical role of CHVs in promoting public health during the COVID-19 pandemic, particularly in Machakos County. CHVs were instrumental in disseminating accurate information on COVID-19 protocols, countering vaccine hesitancy by addressing myths, and mobilizing communities for vaccination campaigns. These successes were facilitated by targeted on-the-job training and close supervision, equipping CHVs with the knowledge and tools to address emerging health challenges. Such findings are supported by other researchers who have demonstrated that adequately trained and well-supervised CHWs significantly improve healthcare delivery and vaccine uptake (Perry *et al.*, 2014). This underscores the necessity of structured training programmes to enable CHVs to meet their roles effectively.

However, we also identified key challenges that hindered the effectiveness of CHVs, including poor remuneration, delayed stipends, insufficient resources, and a lack of recognition and support. These barriers are consistent with other studies that have emphasized the detrimental impact of underfunding and resource shortages on CHW performance during health emergencies (Dahn *et al.*, 2015). Moreover, the financial challenges faced by CHVs were exacerbated by irregular and inadequate payments, which demotivated them and reduced their capacity to fulfil their duties. The issue of delayed and insufficient stipends reflects broader systemic neglect, as highlighted by DeRenzi *et al.*(2017) and Gichaga *et al.* (2021), who attributed such challenges to a lack of institutional appreciation for the role of CHVs in health promotion.

Also, our findings demonstrated that monetary compensation served as a significant enabler of CHV productivity. Timely and adequate payments enhanced their motivation and work output, aligning with other study findings (Kane *et al.*, 2016, Kuule *et al.*, 2017, Ivang and Etienne, 2021). Additionally, Saran *et al.* (2020) and Brunie *et al.* (2014) emphasized that financial incentives improve CHV engagement and performance, reinforcing the importance of addressing remuneration challenges to sustain CHVs’ contributions to public health.

This study reveals a complex dual narrative regarding community relations with CHVs during the COVID-19 pandemic. On one hand, CHVs experienced significant community support and engagement, which facilitated their ability to achieve public health goals, aligning with Glenton *et al.*(2013), who underscored the value of community support in strengthening health worker efficacy. On the other hand, challenges such as social stigma, fear of contracting COVID-19, vaccine hesitancy, and misinformation persisted, mirroring the barriers identified by Sallam (2021) in global vaccine outreach programmes. These contrasting dynamics highlight the critical need for ongoing community sensitization to bridge gaps in trust and understanding of CHVs’ roles.

Priority access to health services emerged as an enabling factor for some CHVs but was inconsistently implemented across Machakos County. This finding corresponds with studies by Asweto *et al.* (2016) and Kuule *et al.* (2017), who emphasized that preferential access to healthcare services for CHWs can enhance performance and morale. Similarly, Kok *et al.* (2017) and Bhaumik *et al.* (2020) identified such non-financial incentives as vital for motivating CHVs and sustaining their commitment to healthcare delivery.

Participation in social support groups was another enabler, with most CHVs belonging to networks such as self-help groups, welfare associations, and table banking circles. These groups fostered emotional resilience, collaboration, and a sense of belonging, as noted by Arora *et al.* (2020) and Saran *et al.* (2020), who recognized social support groups as non-material incentives enhancing CHVs’ work effectiveness. The groups also facilitated trust-building and rapport between CHVs and the communities they served, echoing findings other researchers (Aseyo *et al.*, 2018, Kuule *et al.*, 2017).

Community appreciation of CHVs’ efforts served as a significant motivational factor. Mishra *et al.* (2022) highlighted that recognition of CHWs’ dedication by the community positively influenced their work output. Similarly, studies by Ndima *et al.* (2015) and Musoke *et al.* (2021) affirmed that community acknowledgement of CHVs’ contributions fosters greater dedication and job satisfaction.

However, the study also found limited community support in some areas, potentially due to a lack of awareness about the significance of CHVs’ work. This contrasts with Vareilles *et al.* (2015) and Sharma *et al.* (2014), who reported high levels of social support for CHWs attributed to effective community sensitization. Addressing this gap by enhancing public education about CHVs’ roles could promote stronger community partnerships and bolster CHVs’ capacity to deliver essential health services.

The study highlights significant improvements in the work output of CHVs in Machakos County during the COVID-19 pandemic, as evidenced by increases in household visits, health education sessions, and referrals for care. County health records corroborate these findings, showing substantial gains in key healthcare services such as immunizations, family planning, and antenatal care from 2019 to 2021. The number of children referred for immunization, immunization defaulters tracked, and pregnant women referred rose markedly, from 289 in 2019 to 1,814 in 2021. Similarly, new facility-based deliveries and postnatal visits also surged, reflecting CHVs’ expanded scope of work, including COVID-19-specific roles like vaccine administration, community education, and contact tracing. These findings underscore the essential role of CHVs in maintaining healthcare service continuity during public health crises.

A plausible explanation for these improvements is the increased reliance on CHVs during the pandemic to address gaps in primary healthcare delivery amid strained health systems. During the pandemic, CHVs were recognized as essential service providers, allowing them to continue their work even as government-imposed lockdowns restricted movement for most people. With families largely confined to their homes, CHVs found it easier to establish contact with family units and refer them for necessary services, potentially improving access and outreach compared to the pre-pandemic period. This expanded scope of CHV activities aligns with findings by Razu *et al.* (2021), who noted the critical role of CHVs in supporting health systems by maintaining primary care and implementing COVID-19 countermeasures at the community level. However, contrasting evidence exists. Studies by Rahman *et al.* (2021) and Mishra *et al.* (2022) report a decline in CHW output during the pandemic due to mobility restrictions and limited resources, which hindered basic healthcare delivery. These differences might reflect contextual factors, such as differences in health system support, logistical capacity, and adaptation strategies during the pandemic.

Improved reporting mechanisms during the pandemic, attributed to heightened emphasis on CHVs’ roles, also contributed to these findings. This aligns with Musoke *et al.* (2021), who observed an increased focus on documenting CHW activities during public health emergencies. The implications are profound: strengthening CHV systems, providing adequate support and training, and improving data reporting can enhance healthcare delivery in both routine and crisis contexts.

From the social demographic characteristics of the CHVs we learnt that most were married females aged 30 and above, with basic education backgrounds, and had served as CHVs for over five years. The predominance of females may be attributed to the nature of their work, which focuses on maternal and child health, including family planning, pregnancy, childbirth, vaccinations, and antenatal and postnatal care, aligning with findings from other studies (DeRenzi *et al.*, 2017, Aseyo *et al.*, 2018, Taylor *et al.*, 2018, Mwendwa, 2018, Crispin *et al.*, 2012). The prevalence of basic education among CHVs suggests they primarily addressed fundamental health issues within their communities, referring complex cases to hospitals. Similar findings were reported by other studies where most CHVs had elementary education levels (Aseyo *et al.*, 2018). Further, having served for over five years, most CHVs in Machakos County had substantial experience, equipping them with valuable knowledge of the challenges and enablers of their work. This echoes findings from other studies where many CHVs had substantial years of experience (DeRenzi *et al.*, 2017). Their age (30 years and above) further indicates a maturity that likely enhanced their understanding of their roles in improving community health as also shown by other studies (Crispin *et al.*, 2012, Kuule *et al.*, 2017, Aseyo *et al.*, 2018). Lastly, the fact that most CHVs were in relationships suggests they were well-positioned to understand the health challenges faced by households, supporting findings from other researchers (Crispin *et al.*, 2012, Lister *et al.*, 2017).

### Study limitations

Our study has some limitations. First, we did not conduct a trend analysis or compare data from before and during the COVID-19 period using information from the KHIS. Such an analysis could have highlighted significant differences and allowed for direct attribution of changes to the work of CHVs. Secondly, the gaps or missing data in the reports may have exaggerated some differences/ changes in the presented numbers, requiring cautious interpretation of results that cannot be generalized to other settings. Thirdly, the presence of a PHO as an observer during some FGDs may have influenced participant responses. Despite not directly supervising CHVs, the seniority of the PHO could have shaped how certain information was presented. Fourthly, the involvement of an interviewer who was a nurse and had previously worked within the study community may have unintentionally influenced responses. However, this potential bias was mitigated through methodological triangulation and the use of multiple data sources, ensuring robust findings.

Further, although the study was conducted at the height of the COVID-19 pandemic, subsequent developments in Kenya’s community health programme, such as the rollout of the Primary Health Care Fund and the reclassification of CHVs as Community Health Promoters (CHPs), address some gaps identified in this study. Additionally, initiatives like digital health programmes and social health insurance schemes have further evolved the role of CHVs. As a result, our findings reflect CHVs’ contributions during the pandemic’s peak and do not fully capture the updated dynamics of community health in Kenya. Finally, while it would have been valuable to capture key stakeholders’ opinions, our study did not address this aspect, presenting a gap that future research could explore.

### Policy implications of the study

The findings of this study provide actionable insights for strengthening public health systems by leveraging the pivotal role of CHVs while addressing the systemic challenges they face. The research highlights the need for:

- *Capacity-building initiatives of the CHVs:* Sustained investment in structured training and supportive supervision is critical to equipping CHVs with the skills needed to address diverse and evolving public health challenges effectively.
- *Financial and logistical support:* Timely compensation and adequate resource provision are essential to overcoming barriers such as delayed stipends and insufficient supplies, ensuring CHV morale and operational efficiency are maintained.
- *Integration of CHVs into the health system:* Systematically scaling and institutionalizing CHVs within health strategies will enhance healthcare access and equity, solidifying their role as indispensable components of health systems. This requires equitable workload distribution, enhanced financial support, and formalized recognition of CHVs’ contributions to ensure their sustainability and effectiveness in delivering community health services.
- *Leveraging enablers and addressing barriers:* Strengthening enablers like community recognition and social support while addressing barriers such as social stigma and vaccine hesitancy through public sensitization and non-financial incentives can significantly improve CHV performance.
- *Preparedness for future crises:* Integrating CHVs into national health emergency response plans, supported by investments in training, supervision, and sustainable funding, will enhance their resilience and preparedness for future public health crises.

## Conclusion

In conclusion, this study highlights the indispensable role of CHVs in promoting public health and sustaining healthcare services, particularly during crises like the COVID-19 pandemic. CHVs demonstrated resilience and adaptability, effectively addressing community health challenges through activities such as raising awareness about COVID-19 protocols, dispelling vaccine myths, and mobilizing vaccination efforts. These contributions were bolstered by effective supervision and training, yet hindered by financial and logistical constraints, delayed compensation, excessive workloads, and limited resources. Social dynamics added complexity, with CHVs receiving community appreciation while facing stigma and vaccine hesitancy. Despite these challenges, work output indicators revealed significant improvements in CHV activities, supported by county health records showing marked increases in key health services such as immunizations, antenatal care, and family planning. The findings underscore the need for systemic reforms to enhance financial and logistical support, capacity-building programmes, and community recognition of CHVs. By integrating CHVs into long-term health strategies and addressing barriers to their performance, health systems can ensure their stability and effectiveness in tackling both routine and emergent healthcare challenges. Strengthening community health strategies and sensitizing communities to the critical roles of CHVs will empower these vital workers, transforming them into enduring assets for global health systems.

## Data Availability

All datasets used and analysed during the study are available from the corresponding author at a reasonable request.
